# Contingent National Belonging: The Perceived Fit and Acceptance of Culturally Different Peers Predicts Minority Adolescents' Own Belonging

**DOI:** 10.3389/fpsyg.2018.01975

**Published:** 2018-10-29

**Authors:** Nadya Gharaei, Karen Phalet, Fenella Fleischmann

**Affiliations:** ^1^Faculty of Psychology and Educational Sciences, Center for Social and Cultural Psychology, University of Leuven, Leuven, Belgium; ^2^Ercomer, Department of Interdisciplinary Social Science, Utrecht University, Utrecht, Netherlands

**Keywords:** minority adolescents, cultural difference, national identification, national identity content, fit, peer context, social belonging

## Abstract

Prevailing definitions of national identities in Europe equate belonging to the nation with “fitting in” culturally and leave immigrant minorities who are culturally different from the majority group struggling to belong. The present study focuses on an under-researched minority perspective on the intersubjective cultural contents of the national identity. We propose that minorities' national belonging is contingent on their perception that minority peers who deviate from the majority culture are accepted as real nationals. Our study aims to establish (a) minority perceptions of the national fit and acceptance of culturally different peers, and to test (b) the consequences of perceived fit and acceptance for minority adolescents' own national belonging, and (c) its affordances by the local peer context. Drawing on a large random sample of 1,489 Moroccan and Turkish minority youth (aged 12–18) and their peers across 312 classes in 63 Belgian schools, we varied cultural difference from the majority in three vignettes describing imaginary acculturating peers. Minority participants rated to what extent they saw each peer as a real national (perceived fit) and whether other nationals would accept this peer (perceived acceptance). As a measure of their own national belonging, they indicated their national self-identification. Additionally, the multi-level design included classroom contextual measures of majority peer presence and peer acculturation norms (peer norm of heritage culture maintenance). As expected, minority youth who perceived better national fit of culturally different peers, self-identified more strongly as nationals than those who perceived worse fit. This association was not explained by their own acculturation attitudes. In line with the contextual affordance of national fit, only in classes with majority peers, minority youth perceived higher national fit and acceptance of culturally different peers when peer norms supported the maintenance of a distinct heritage culture. We conclude that the national belonging of minority youth is contingent on the peer context through the perceived fit and acceptance of culturally different peers.

## Introduction

Imagine Fatma. Fatma is 14 years old and born in Belgium from Turkish immigrant parents. Her best friends are Turkish and she wants to marry a Turkish boy later. She loves Turkish food and likes to go on holidays to Turkey. While Fatma's Turkish heritage culture makes her different from her majority Belgian peers, they attend the same Belgian school together and they are all future adult Belgian citizens. Our research focuses on the degree to which minority adolescents perceive acculturating peers like Fatma to be true Belgians. We propose that minority adolescents' own national belonging will be contingent on their perception that culturally different peers like Fatma (mis)fit with the national identity.

Social belonging is a human need essential for psychological well-being and success (Baumeister and Leary, 1995). For instance, a sense of belonging in adolescents was shown to promote academic motivation and achievement (Booker, 2004; Faircloth and Hamm, 2005; Walton and Cohen, 2011; Gillen-O'Neel and Fuligni, 2013), to reduce internalizing and externalizing problem behaviors (Newman et al., 2007; Pittman and Richmond, 2007), and to even improve health (Walton and Cohen, 2011). One key source of social belonging that gains in importance during adolescence is the national group (Barrett, 2000). As minority adolescents venture out into wider social circles beyond the family, their relations with minority as well as majority peers play a key role in the development of a sense of national belonging.

One less studied way in which peer relations afford national belonging (or not), is through informing minority adolescents' perceptions of “fit” with the national identity. At the heart of a distinct minority perspective on what it means to be a national is the articulation of cultural difference from the majority. Because of their cultural minority status, minorities' fit with the national identity is essentially contested and depends crucially on signals of acceptance from other nationals (Verkuyten and Yildiz, 2007; Fleischmann and Phalet, 2016, 2018). Thus, minority adolescents have to negotiate their national fit in social interactions with minority as well as majority peers in order to belong (Umaña-Taylor, 2004). In most European countries, prevailing definitions of the national identity equate belonging to the nation with “fitting in” culturally (Phalet and Kosic, 2006). Consequently, immigrant minorities are expected to ideally become culturally indistinguishable from majority nationals. In contrast, minorities most often prefer to combine the majority culture with *some* maintenance of a distinct heritage culture (Phinney et al., 2001; Verkuyten and Martinovic, 2012). Therefore, definitions of the national identity referring exclusively to the majority culture, leave minorities struggling to belong (Van Acker, 2012; Reijerse et al., 2013). Accordingly, immigrant minorities across Europe report significantly less belonging to the national group than majorities (Phinney et al., 2006; De Vroome et al., 2014; Fleischmann and Phalet, 2018).

Against this background, our main study aim is to investigate minority adolescents' perceptions of national identity content as an explanation for their national belonging. Taking an intersubjective approach, we conceive identity contents as shared understandings of what it means to be a real national (Chiu et al., 2010). Our focus here is on the under-researched cultural contents of the national identity. For our purposes, we are specifically interested in whether these contents refer exclusively to the majority culture, or whether they leave some room for contributions from minorities' distinct heritage cultures. To this end, we assess the cultural contents of the national identity indirectly by asking participants to what extent imagined peers who vary in their degree of cultural difference from the majority group are true nationals (perceived fit) and accepted by other nationals (perceived acceptance). The aims of our study are threefold. As a novel way to gauge minority views on the national identity, we assess their perceptions of national fit and acceptance of peers who are culturally different from the majority (aim 1). Next, we examine how perceiving culturally different peers to fit with the national identity relates to minority adolescents' own national belonging (aim 2). Finally, we ask when the peer context in culturally diverse classrooms affords the perception of national fit and acceptance of culturally different peers (aim 3).

To address these aims, we draw on large random samples of 1,489 Moroccan and Turkish minority youth (aged 12–18 years) and their peers in 312 classes across 63 secondary schools in Belgium (Emonds et al., 2014). Minority status is defined here by adolescents' self-reported foreign-born parentage (i.e., they themselves, their parents, or their grandparents were born in Turkey or Morocco). As major Muslim minorities in Europe, Turkish and Moroccan immigrant workers are among the most devalued and disadvantaged immigrant groups in Europe (Voas and Fleischmann, 2012; Heath and Brinbaum, 2014) and are hence least likely to be accepted as real nationals. As part of the survey, participants rated the perceived national fit and acceptance of imagined minority peers in three vignettes which varied their cultural difference from the majority (aim 1). In addition, participants reported (*inter alia)* their national self-identification as a measure of their own belonging (aim 2). Finally, the multi-level study design with minority participants nested in culturally diverse classrooms adds measures of the actual peer context (aim 3). Despite the nominal minority status of Turkish and Moroccan origin youth in the wider society, they are often a local numerical majority in their diverse classrooms. The highly ethnically segregated and stratified structure of many European school systems is well-documented, in particular in Belgium (Baysu and de Valk, 2012). As a consequence, majority-minority and even all-minority peer contexts are an increasingly common yet under-studied social reality of many minority youth in European societies. Due to this social reality, the present study compares those in all-minority classrooms to those who do have a majority peer presence in class.

Our research adds to the social-psychological literature in a number of ways. First, research on national identity mostly examines how strongly people identify with the national group without articulating the meaningful contents of the national identity (Ashmore et al., 2004; Eugster and Strijbis, 2010). Hence, the finding that minorities are less strongly identified as nationals than majorities begs the question what the national identity means for them (Verkuyten and Martinovic, 2012). Moreover, the few studies of national identity contents were mainly limited to majority group perspectives (e.g., Pehrson et al., 2009; Pehrson and Green, 2010; Duriez et al., 2013). A cross-national analysis of national identity contents among majority youth in six European countries, for instance, documented majority group cultural institutions, practices, and values as contents of a “cultural representation” of the national identity (Reijerse et al., 2013). Though little is known about minority perspectives, a study of minority representations of the Belgian national identity revealed similar associations with majority cultural values and institutions (Phalet and Swyngedouw, 2002).

Moreover, we add to an intergroup relations approach to national identification by zooming in on the peer relations of minority adolescents. Extensive research has related minority national identification to individual experiences of culture contact and acculturation attitudes on the one hand (Ashmore et al., 2004; Umaña-Taylor et al., 2014; Verkuyten, 2014) and to perceptions of intergroup relations in the wider society on the other hand (Verkuyten and Yildiz, 2007; Mähönen and Jasinskaja-Lahti, 2012; Martinovic and Verkuyten, 2012; Wiley, 2013). In parallel, an emerging stream of research on minority peer relations has yielded mixed findings of positive, zero, or reverse effects of majority peer presence and contact on minority national identification (Agirdag et al., 2011; Leszczensky et al., 2016).

Extending previous research, our study foregrounds minority perceptions of national fit and acceptance as connecting processes between minority national belonging and the peer context. We relate perceived national fit and acceptance to minority as well as majority peer relations. One reason to include minority peers as part of the actual peer context is their numerical presence in culturally diverse classrooms. Another reason is that the generational status of mostly second-generation (local-born rather than foreign-born) minority youth in our sample formally entails full national membership. Hence, minority as well as majority peers jointly represent the common national identity in culturally diverse classrooms. Nevertheless, intergroup relations between immigrant minority and majority nationals in the wider society are unequal, so that the majority group most powerfully defines the national identity. Minority definitions of the national identity may hence need to be socially verified by *some* member(s) of the nominal majority. To take into account such macro-constraints on the definition power of minorities, our study tests the absence of majority peers as a necessary condition on peer affordances of national fit and acceptance.

### Minority perceptions of national fit and acceptance

Our first research aim is to establish minority perceptions of national fit and acceptance. More precisely, we asked minority youth how they view acculturating peers like Fatma: Do they see her as a true Belgian? Do they expect other Belgians to accept her? These questions reflect our conceptual approach to the national identity from the intersubjective nature of its cultural contents (Chiu et al., 2010). Intersubjective beliefs are distinct from personal beliefs and refer to socially shared understandings. *In casu*, intersubjective beliefs refer to the cultural attributes that define a real national (Ashmore et al., 2004). Such beliefs are socially verified in ongoing interactions with significant others within one's social environment (Chiu et al., 2010). I*n casu*, national identity definitions are verified by the peer context in culturally diverse classrooms. In keeping with the generic normative nature of category fit (Voorspoels et al., 2011), the cultural attributes of a real national are not necessarily widespread or frequently observed; rather, they refer to an ideal cultural instantiation of the national identity. When such cultural ideals are shared by many or most nationals we know, they acquire the psychological quality of an objective reality (Higgins, 2016). Accordingly, we propose that the intersubjective nature of national identity contents imposes real constraints on minority perceptions of national fit and acceptance. For instance, when intersubjective definitions of the national identity refer exclusively to the majority culture, minority adolescents may *not* see Fatma as a real national to the extent that she maintains her distinct heritage culture.

To the extent that intersubjective beliefs are negotiated in social interactions, national identity definitions need to be verified by other nationals within the social context (Swaab et al., 2007). Thus, prevailing definitions of the national identity can be challenged by alternate views from minority nationals in particular (see the notion of national identity heterogeneity; Falomir-Pichastor and Frederic, 2013). Along those lines, minority youth actively co-construct what it means to be a real national in interactions with minority as well as majority peers in culturally diverse peer contexts (Rutland et al., 2011). For minority adolescents to see Fatma as a real national, for instance, it may be required that the actual peer context affords national fit and acceptance of minority nationals who are culturally different from the majority.

In keeping with the intersubjective nature of national identity contents (Ashmore et al., 2004; Chiu et al., 2010), we assessed the perceived national fit of acculturating peers—rather than minority adolescents' own national fit. Specifically, we asked to what extent minority participants view peers who are culturally different from the majority as true nationals. For instance, do they see Fatma, who maintains distinct Turkish cultural customs, as a real Belgian? To find out, we varied the degree of cultural difference from the majority group across three vignettes describing imaginary peers with different acculturation attitudes. In line with Berry's (1997) bi-dimensional model of acculturation, the separated peer displayed high heritage culture maintenance and low majority culture adoption, and was therefore *most* culturally different from the majority group. *Less* culturally different from majority nationals was the integrated peer, who was oriented toward both heritage and majority cultures. Finally, *least* culturally different was the assimilated peer, who displayed high majority culture adoption and low heritage culture maintenance. To assess perceived national fit and acceptance as a function of cultural difference from the majority group, we asked minority participants whether they perceived the imagined acculturating peers in the vignettes as real Belgians and whether they would be accepted by other Belgian nationals.

### From perceived fit and acceptance to own national belonging

The second research aim concerns the psychological implications of the perceived national fit and acceptance of acculturating peers for minorities' own national belonging. One way for minorities as individuals to fit with the national identity, and hence to achieve national belonging, is to individually assimilate to the majority culture. In line with prevailing assimilationism in Europe, majorities tend to view adopting the majority culture as incompatible with maintaining the heritage culture (Verkuyten and Martinovic, 2012). In this vein, Verkuyten and Thijs (2002) demonstrated that majority Dutch adolescents expected minorities to relinquish their heritage culture in order to adopt the majority culture. Similarly, majority Belgian youth who were told that immigrants maintained the heritage culture more, inferred that they adopted the majority culture significantly less (Van Acker and Vanbeselaere, 2012).

When they fully adopt the majority culture, immigrant minorities become less culturally distant or distinct from majority nationals. Thus, they conform to a prevailing definition of the national identity in majority cultural terms. In support of this assimilationist pathway to national belonging, majority Belgians evaluated minority peers more positively when they adopted the Belgian culture, and this positive association was mediated by an increase in majority perceptions of their national belonging (Roblain et al., 2016).

Research has shown, however, that this way to achieve national belonging is psychologically costly for minority youth. By relinquishing their heritage culture, minorities lose known social and psychological benefits of heritage culture maintenance, such as social support, self-worth, and psychological health and wellbeing (Berry et al., 2006; Schachner et al., 2014). Moreover, even fully assimilated minority persons can still be rejected by majority nationals in less welcoming acculturation contexts or in the presence of visible minority status (Van Acker, 2012).

Looking beyond a well-established pathway from individual assimilation to national identification, our study examines an alternate pathway to national identification through the perceived national fit and acceptance of acculturating peers. More generally, experimental evidence suggests two complementary cognitive processes called self-stereotyping and self-anchoring. Both processes were shown to enable the self-identification of group members by mentally connecting the self and the group albeit in distinct ways (Jans et al., 2012; Van Veelen et al., 2016). “Self-stereotyping” is a well-known cognitive pathway to group identification that derives from Social Categorization Theory (SCT; Turner et al., 1987) and entails defining the self by aligning its attributes with the prevailing stereotype of a real or ideal group member (Lafrora et al., 2010). Applied to the national identity, minority individuals who self-stereotype as nationals align the self with the definition of an ideal national member in majority cultural terms. This process of self-stereotyping entails that they adopt majority cultural attributes while dissociating the self from heritage cultural contents that do not fit with this definition. Along those lines, minorities in Europe who prefer to adopt the majority culture more, and to maintain the heritage culture less, are most strongly identified with the nation (Verkuyten and Martinovic, 2012). Our study takes into account this well-documented assimilation-identification pathway by including minorities' own acculturation attitudes toward mainstream culture adoption and heritage culture maintenance as predictors of their national identification.

In addition to self-stereotyping, an alternate cognitive pathway to group identification has been called “self-anchoring”: this process creates a mental link from the self to the group. More precisely, self-anchoring denotes defining the group by highlighting group attributes that are in line with core aspects of the personal self (Cadinu and Rothbart, 1996; Van Veelen et al., 2016). Recent experimental evidence confirms that not only self-stereotyping but also self-anchoring enables the self-identification of group members (Van Veelen et al., 2016). Applied to the national identity, self-anchoring entails mentally connecting self-relevant heritage cultural contents to the national identity. Most minorities place importance on their heritage culture as part of who they are; as such, it is a core aspect of their personal self. Not only do they feel proud of their heritage culture, maintaining aspects of it also protects their personal self-esteem (e.g., Phinney et al., 1997; Umaña-Taylor, 2004; Verkuyten and Thijs, 2004)^1^. When minorities self-identify as nationals through self-anchoring, they define the national identity so that it includes self-relevant heritage cultural contents as well. From an intersubjective approach to the cultural contents of the national identity, self-anchoring requires that minorities perceive some degree of national fit in the presence of cultural difference from the majority. Our study thus examines a novel pathway to national identification through self-anchoring by assessing minority perceptions of national fit and acceptance of culturally different peers. This hypothetical pathway complements a parallel pathway to national identification through the individual assimilation of minority youth. Importantly, self-anchoring avoids the psychological costs of dissociating heritage cultural aspects of the self to fit into an elusive majority cultural ideal of national membership. Applying self-anchoring to the national identity, therefore, we hypothesize that minority youth who perceive culturally different peers to fit the national identity better, will self-identify more strongly as nationals than those who perceive worse fit (Hypothesis 1).

As cultural identity contents of the national identity reflect shared understandings of what it means to be a real national (Ashmore et al., 2004; Verkuyten and Martinovic, 2012), they need to be verified in social interactions with other nationals. Though national identity definitions depend crucially on social acceptance by majority nationals, minority nationals can also add weight to alternate identity definitions (Modood, 2007). A qualitative study shows, for instance, that Turkish origin young adults in Austria actively co-construct shared understandings of the national identity; and they negotiate heritage and majority cultural contents of their Austrian identity in social relations with other Austrians (Vietze et al., 2018). We therefore expect that minority adolescents will perceive better national fit of culturally different peers, when they perceive that these peers will be socially accepted by other nationals (Hypothesis 2).

### Contextualizing perceived fit and acceptance

The third and final study aim concerns the affordances of perceived national fit and acceptance in the actual peer relations of minority adolescents. In addition to the perceived fit and acceptance of imagined peers in the vignettes, the multi-level design of our study brings in the actual day-to-day peer context of minority adolescents in culturally diverse classrooms. Peer influences are at their peak during adolescence (Brown and Larson, 2009); and the norms that peers communicate are an important source of information and influence (Aboud and Fenwick, 1999). Applied to acculturation norms, Thijs and Verkuyten (2013) found that Dutch adolescents' own acculturation attitudes were informed by the acculturation attitudes of their classmates. Similarly, Celeste et al. (2016) predicted the social acceptance of minority adolescents from the acculturation norms of their classmates. Along those lines, we expect that acculturation norms in diverse classrooms, in particular whether (actual) peers value the maintenance of a distinct heritage culture, will inform the perceived acceptance of (imagined) peers who are culturally different from the majority (Hypothesis 3).

Finally, due to the highly stratified and segregated school system in Belgium (Merry, 2005; Baysu and de Valk, 2012), minority youth often find themselves in classrooms with only few majority peers or none. While minority as well as majority peers can inform minority perceptions of national fit and acceptance, we do not know whether minority peers can define the national identity in the absence of social verification by majority peers. In the absence of majority peers, the local peer context in the classroom is disconnected from the national majority group who powerfully define the national identity in the wider society. Peer norms in all-minority classrooms may therefore fail to generalize beyond the local peer context. Therefore, we additionally explore whether *some* majority peer presence in class is a necessary condition for Hypothesis 3 to hold.

### Overview of the study

To summarize, the present study investigates a distinct minority perspective on national belonging in culturally diverse peer contexts. Its starting point are the intersubjective cultural contents of the national identity. We reason that identity definitions which exclusively refer to the majority culture may complicate national belonging for minorities by undermining their perceptions of national fit and acceptance in the presence of cultural difference from the majority. To put our reasoning to a test, our study aims to, first, assess minority adolescents' perceptions of national fit and acceptance of (imagined) acculturating peers who are culturally different from the majority. Second, we relate these perceptions of national fit and acceptance to minority adolescents' own national belonging. In line with a less well researched pathway to national identification through “self-anchoring,” we predict that minority youth who perceive culturally different peers to fit the national identity better, will self-identify more strongly as nationals than those who perceive worse fit (Hypothesis 1), and will also perceive more acceptance of culturally different peers by other nationals (Hypothesis 2). Finally, we examine the affordance of perceived national fit and acceptance by the actual peer context in culturally diverse classrooms. Specifically, we expect that minority youth will perceive more national fit and acceptance of culturally different peers, when local peer norms in class support heritage culture maintenance (conditional on majority peer presence; Hypothesis 3).

## Methods

### Data

We used large-scale survey data from the Leuven-CILS project in culturally diverse lower secondary schools in Flanders, Belgium. This project is modeled on and affiliated with the Children of Immigrants Longitudinal Survey in Europe (CILS4EU; Kalter et al., 2014); and it combines self-report measures of minority adolescents' national self-identification and acculturation with contextual classroom measures (e.g., of peer norms) in a multi-level design (Emonds et al., 2014 for detailed information on the sample and the complete constructs and measures). Using school-level administrative information on foreign languages spoken at home, *n* = 70 schools with varying shares of immigrant minority students (< 10%, 10–40%, 40–60%, >60%) were selected in line with the CILS4EU stratified random sampling design. Prior to the administration of paper-and-pencil questionnaires, informed consent from school principals, teachers, participants and their parents was obtained. Participation was voluntary (students could drop out at any time) and anonymity was guaranteed. Students from randomly sampled classes in grades 1–3 filled out the two-part questionnaire in two consecutive class hours in the presence of trained research assistants and a teacher.

### Participants

For the analysis, we selected Moroccan and Turkish minority youth from the total sample of *N* = 5336 students in 70 schools on the basis of self-reported parentage (i.e., those with at least one parent or two grandparents born in Morocco or Turkey). Our final sample for analysis consisted of *N* = 1489 minority adolescents of Moroccan (*N* = 834) and Turkish origin (*N* = 655) in *n* = 312 classrooms across 63 secondary schools in Flanders, Belgium. Moroccan and Turkish minority youth are pooled for the analysis, because they share similar migration histories as (grand)children of immigrant workers from majority-Muslim countries; and they face similar levels of persistent disadvantage and pervasive prejudice in Belgian and European intergroup contexts (Strabac and Listhaug, 2008; Heath and Brinbaum, 2014). The vast majority of the Moroccan and Turkish minority participants were 2nd generation immigrants (1st generation *N* = 174, 2nd generation *N* = 1202, 3rd generation *N* = 122)^2^ and self-categorized as Muslim (83%); their ages ranged from 12 to 18 (*M*_age_ = 15.06, *SD* = 1.22), and 47% were female^3^. To ensure the reliability of contextual measures in multi-level models, which are sensitive to outliers, we excluded from the analysis those in classrooms with < 5 students (*N* = 75). Next, we excluded students with no migration background (whose parents and grandparents were all born in Belgium; *N* = 2047), and those with an immigrant background other than Moroccan or Turkish (*N* = 1722). As our study targets early to mid-adolescents in lower secondary schools, we additionally excluded at the individual level of analysis three participants over 18 years of age. At the classroom level, we used data from majority, Moroccan, Turkish and other minority peers (*N*_*total*_ = 5261) to compute our contextual measures of the actual peer context.

Finally, three vignettes were embedded within the multi-level design of the CILS survey, specifically targeting Moroccan and Turkish minority participants. The three vignettes featured three imaginary new Muslim minority classmates with different acculturation attitudes (indicative of the degree of cultural difference from the majority group). The vignettes are modeled on similar vignettes that have been used to evoke distinct acculturation attitudes in experimental acculturation research (cf. Brown and Zagefka, 2011; Van Acker and Vanbeselaere, 2012 for a Belgian example). Unlike previous research, however, we use the acculturation vignettes as a novel way to assess the perceived national fit and acceptance of imaginary peers as a function of their cultural difference from the majority group (cf. infra).

### Measures

#### National self-identification

A sense of belonging to the national group was measured with the question “How strongly do you feel Belgian?” rated on a (reverse coded) scale ranging from 1 (I don't feel Belgian) to 5 (very strongly)^4^. This measure taps into commitment or attachment to a group as a key affective aspect of collective identification (Phinney, 1992; Ashmore et al., 2004). The mean of our measure of minority adolescents' national self-identification was not significantly different from the scale midpoint (*M* = 2.95, *SD* = 1.37; 3 means “not so strongly”).

#### Perceived national fit of culturally different peers

To assess the perceived national fit of minority peers who are culturally different from the majority, cultural difference was varied between three vignettes describing imaginary peers. The perceived national fit of imaginary culturally different peers was measured with the question “Do you think that … is a real Belgian?” Participants answered the question on a five-point scale (reverse coded: 1 = absolutely not, 5 = very much). Responses indicate to what extent they perceived the imagined peers to fit with the national identity.

More precisely, participants read three vignettes describing imaginary new minority classmates with different acculturation attitudes of separation, integration and assimilation (Berry, 1997). As the wordings of the vignettes in Table 1 show, the separated peer (Fatma) is *most* culturally different from majority Belgians as she is maintaining the customs of her heritage culture while not adopting the customs of the majority culture. *Less* culturally different than Fatma is the integrated peer (Ayse), who is oriented toward both the majority and her heritage culture. Lastly, the assimilated peer (Azize) is the *least* culturally different of the three as she is described as having adopted customs of the majority culture while not maintaining the customs of her heritage culture. Gender was kept constant across the vignettes; similarly common Muslim names were used and specific cultural contents were slightly different between the vignettes with a view to enhancing face validity in a within-subject design. Note that we measured the perceived national fit of imagined peers, rather than minorities' self-perception of their *own* national fit; thus, our measure avoids self-representation biases (Hughes and Huby, 2002) and resonates with the intersubjective conceptual approach (Chiu et al., 2010).

**Table 1:** Vignettes, means, and standard deviations of *perceived national fit* and *acceptance of (imagined) cultural different peers*.

**Vignettes Imagine {Fatma/ Ayse/ Azize} being a new student in your class. {Fatma/ Ayse/Azize} is 14 years old. Her parents moved from Turkey to Belgium before she was born. …**		***Perceived national fit of culturally different peers***		***Perceived acceptance of cultural different peers***	
		**Measures**	***M* (*SD*)**	**Measures**	***M* (*SD*)**
Separated	Fatma feels fully Turkish and little Belgian. She only has Turkish friends and wants to marry a Turkish boy later. She prefers Turkish food over Flemish food and likes to watch Turkish movies. She doesn't watch Belgian television. Fatma wears a head scarf and loves to go on holidays to Turkey.	“Do you think that Fatma is a real Belgian?”	2.00 (1.30)^a^	“Do you think that most Belgians like Fatma?”	3.00 (1.22)^a^
Integrated	Ayse feels Belgian. She has lots of Belgian friends and is part of a youth movement. She really likes to eat Belgian food like French fries and chocolate. But Ayse also feels Turkish. She likes to speak Turkish with other Turks and she really likes to drink Turkish tea. She also loves Turkish music and would like to wear a head scarf later.	“Do you think that Ayse is a real Belgian?”	2.59 (1.27)^b^	“Do you think that most Belgians like Ayse?”	3.46 (1.09)^b^
Assimilated	Azize feels fully Belgian and little Turkish. She only has Belgian friends and is in love with a Belgian boy from her class. She likes to watch Flemish television shows and likes to read comic books in Dutch. Azize speaks better Flemish than Turkish and knows little about the Islam.	“Do you think that Azize is a real Belgian?”	3.40 (1.36)^c^	“Do you think that most Belgians like Azize?”	4.01 (1.07)^c^

*All measures were rated on a 5-point scale (reverse coded: 1 = absolutely not, 5 = very much). The term “Flemish” in the vignettes refers to the Dutch language and customs in the Dutch-speaking part of Belgium (known as Flanders or the Flemish Region) were data collection for this study was conducted. Significant mean differences (at p < 0.001) within columns are indicated by use of different superscripts*.

#### Perceived acceptance of culturally different peers

In addition to perceived national fit, the same vignettes were also used to measure the perception that other Belgian nationals are accepting peers who are culturally different from the majority. Specifically, minority participants answered the question: “Do you think that most Belgians like {Fatma/ Ayse/ Azize}?” (again on a five-point scale; reverse coded: 1 = absolutely not, 5 = very much).

#### Peer norm of heritage culture maintenance

To assess peer acculturation norms with regard to heritage culture maintenance, we asked a commonly used general question measuring peer attitudes toward immigrant minorities maintaining their heritage culture (Arends-Tóth and van de Vijver, 2006). Specifically, all (minority and majority) peers in each class (*N*_total_ = 5261) were asked to rate the following statement: “Migrants should do everything possible to preserve the customs of their country” on a (reverse coded) five-point scale ranging from 1 (strongly disagree) to 5 (strongly agree). To compute a contextual peer norm measure, individual responses were aggregated over all classmates—including majority as well as Moroccan, Turkish and other minority peers within each class, and excluding only the minority participant's own attitude toward heritage culture maintenance. The mean peer norm of heritage culture maintenance across classrooms was 3.29 (*SD* = 0.44), which is significantly above the scale midpoint [*t*_(1488)_ = 21.90, *p* < 0.001]. This suggests that overall, peer norms in our culturally diverse classrooms tend to accept heritage culture maintenance.

#### Majority peer presence

The presence of majority peers was computed as the relative proportion of peers in class whose parents and grandparents were born in Belgium (range 0–1 with 1 referring to all other peers in class including other minorities than Moroccan or Turkish as well). Due to school segregation, 35% of all Moroccan and Turkish minority participants in our study had no majority peers in their class. Highly diverse classrooms with very few or no majority youth are increasingly common in today's schools across Europe as a consequence of ethnic segregation and continuing immigration (Baysu and de Valk, 2012; Celeste et al., 2016). Yet, we know very little about the acculturation and identification of minority youth in the absence of majority peers. From a theoretical viewpoint, it is essential to empirically establish the generalizability of our findings to under-researched all-minority environments. Therefore, the present study tests majority peer presence as a necessary condition for the expected effects of local peer norms.

To compare local peer contexts with and without a majority presence, additional multi-level analyses were conducted separating a subsample of 35% of our minority participants in all-minority classrooms from the remaining 65% in classes with majority peers. In the latter subsample, the mean share of majority peers across classes was 0.36 (*SD* = 0.23). This suggests that minority participants in our study were a local numerical majority in most classes. Note that most classrooms were culturally diverse even in the absence of majority peers, including other than Turkish or Moroccan minority peers as well. Thus, average shares of Moroccan and Turkish minority peers in classrooms without and with a majority peer presence were 0.64 (*SD* = 0.22) and 0.34 (*SD* = 0.23) respectively. Other minority peers in the classes (*N*_total_ = 1722) originated from a wide range of other countries (> 100 origins).

#### Control variables

In order to estimate net effects of the main predictor variables, we included *ethnic origin* (1 = Turkish, 0 = Moroccan) and *gender* (1 = girls, 0 = boys) as statistical controls for each association of interest in our models. In addition, we controlled for associations of minority adolescents' own acculturation attitudes with their national self-identification. Specifically, participants' *own attitude toward heritage culture maintenance* was assessed using the statement as described for our contextual peer norm measure, while their *own attitude toward majority culture adoption* was assessed with the statement: “Migrants should adopt the Belgian customs in this country” (reverse coded: 1 = strongly disagree, 5 = strongly agree). Age as a control was omitted from the final analysis as it did not have any significant effects.

#### Analyses

To address our first research aim, we explore minority perceptions of the national fit and acceptance of peers who differ culturally from the majority. To this end, we compare the means of our perceived national fit and acceptance measures across the vignettes—namely, the perceived fit and acceptance of the (imagined) separated, integrated and assimilated peers.

The main analyses consist of multi-level regressions in Mplus 7 (Muthén and Muthén, 1998-2012). All regression analyses were controlled for ethnic origin, gender and participants' own acculturation attitudes. We accounted for the nested structure of our data by specifying school classes as clusters (*n* = 312). In a first step of the analyses, we address our second research aim about the association of minorities' own national belonging with the perceived national fit and acceptance of culturally different peers. In order to test Hypothesis 1 and 2 about the role of perceived national fit and acceptance, respectively, we estimated a multi-level path model with national self-identification as the dependent measure, the perceived national fit of (imagined) culturally different peers as mediators, and their perceived acceptance by other nationals as predictors. All associations were specified at the individual level; three separate paths were estimated regressing national self-identification on our three measures of perceived national fit, and on the corresponding measures of perceived acceptance in the three vignettes with separated, integrated and assimilated peers. Although our main interest here lies in the within-vignette associations of national self-identification with the perceived fit and acceptance of the culturally different peers, we also estimated the (six) remaining associations across vignettes^5^ for completeness. Finally, non-significant direct paths from perceived acceptance to national self-identification were removed from the final model.

In a second step of the multi-level regression analyses, we address our last research aim about the affordance of perceived national fit and acceptance by the peer context in culturally diverse classrooms. To test Hypothesis 3 about the role of peer norms of heritage culture maintenance, we added our contextual measure of the peer norm to predict the perceived acceptance of the (imagined) culturally different peers in the vignettes. Note that technically, peer norms of heritage culture maintenance was specified at the individual level, because taking out the individual maintenance attitude of each participant resulted in slightly different aggregate scores for each participant within the same class. Moreover, we empirically tested the absence of majority peers in all-minority classrooms as a necessary condition on the association of peer norms with perceived acceptance; to this end, we distinguished between minority participants who were in classrooms with some majority peer presence (*N* = 968) and those who were in classrooms with no majority peers (*N* = 521). To test for possible differential effects between the two subsamples, we specified a multi-group multi-level model and formally tested equality constraints on theoretical effects using Wald Chi-square tests (Muthén and Muthén, 1998-2012).

Finally, all models were based on the pooled sample of Moroccan and Turkish minority youth—controlling for ethnic origin—with a view to increase statistical power and to ensure a reasonable distribution of individual participants over all classrooms as contextual units in multi-level modeling. However, additional analyses (see sub-section on robustness checks) replicated our main results by imposing equality constraints on theoretical effects in a multi-group model distinguishing between Moroccan (*N* = 834) and Turkish (*N* = 655) subsamples.

## Results

### Minority perceptions of national fit and acceptance

Descriptive results address our first research aim to establish minority perceptions of the national fit and acceptance of peers who differ culturally from the majority. Table 1 shows that Moroccan and Turkish minority participants defined the imagined “separated” peer least strongly as a real Belgian, indicating low national fit. The mean for the imagined “integrated” peer was still significantly below the midpoint of the five-point scale [*t*_(1427)_ = −12.32, *p* < 0.001], again indicating relatively low national fit in the eyes of minority participants. Only the “assimilated” peer in the vignettes was defined as a real Belgian, with a mean score above the midpoint of the scale [*t*_(1433)_ = 11.01, *p* < 0.001]. However, the mean score indicates that minority participants perceived only moderate national fit, suggesting that even assimilating does not make you entirely or unambiguously a real Belgian. As expected from prevailing definitions of the national identity in majority cultural terms, minority youth perceived separated or integrated peers, who are most culturally different from the majority, to fit the national identity less well than a least different assimilated peer.

Similarly, also reported in Table 1, minority participants perceived Belgian nationals to be least accepting of the separated, and most accepting of the assimilated imaginary peer, with the acceptance of the integrated peer in-between. The mean acceptance of the most culturally different (separated) peer was not significantly below the scale midpoint (3 means “a little bit”). This suggests that minorities perceive ambivalent or uncertain acceptance—rather than outright rejection by other Belgian nationals.

### From perceived fit and acceptance to own national belonging

**Figure 1:**
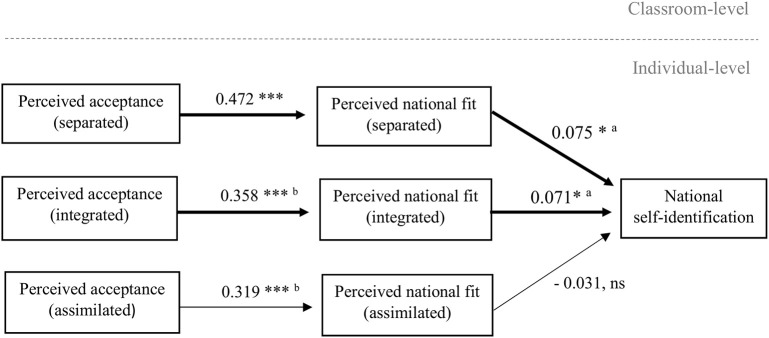
Multi-level path model with national self-identification as the final dependent variable while controlling for student nested in school classes. Standardized regression coefficients are reported, and significant indirect paths are shown with arrows in bold. Identical superscripts indicate effects that did not significantly differ. Full model results, including effects of control variables, are reported in Table 2. ^***^p < 0.001, ^*^p < 0.05, ns = not significant at *p* = 0.292.

The first multi-level regression model addresses our second research aim, which is to explain minorities' own national belonging from the perceived national fit and acceptance of the (imagined) culturally different peers. Respective model results, while controlling for students nested in school classes, are presented in Table 2 and visualized in Figure 1. Standardized regression coefficients and 95% confidence intervals are reported. Our findings show that the perceived national fit of the imagined separated and integrated peers were both positively related to Moroccan and Turkish minority adolescents' self-identification as Belgian nationals, while no significant relation with national self-identification was found for the perceived national fit of the imagined assimilated peer. Moreover, the former two significant effects did not differ from each other (Wald χ^2^(1) = 0.002, *p* = 0.966), suggesting that the perceived national fit of the separated peer and the integrated peer promoted national self-identification to the same extent. These results support Hypothesis 1, namely that minority adolescents who perceive integrated or separated peers to fit the national identity better, self-identify more strongly as nationals than those who perceive worse fit.

Table 2Multi-level path model with national self-identification as the final dependent variable and while controlling for students nested in school classes.

**Table:** 

**Effects on national self-identification**	**β**	**CI**
Perceived national fit (separated)	0.075^*^^a^	[0.011; 0.139]
Perceived national fit (integrated)	0.071^*^^a^	[0.009; 0.133]
Perceived national fit (assimilated)	−0.031^∧^	[−0.089; 0.027]
Own attitude toward maintenance	−0.088^**^	[−0.146; −0.029]
Own attitude toward adoption	0.228^***^	[0.167; 0.288]
Girls	0.104^***^	[0.046; 0.162]
Turkish	0.092^**^	[0.033; 0.152]

**Table:** 

**Effects on perceived national fit**	**Separated**		**Integrated**		**Assimilated**	
	**β**	**CI**	**β**	**CI**	**β**	**CI**
Perceived acceptance (separated)	0.472^***^	[0.421; 0.522]	0.075^*^	[0.013; 0.137]	−0.056	[−0.116; 0.004]
Perceived acceptance (integrated)	0.023	[−0.036; 0.081]	0.358^***^^b^	[0.295; 0.421]	−0.009	[−0.072; 0.054]
Perceived acceptance (assimilated)	−0.128^***^	[−0.182; −0.074]	−0.087^**^	[−0.141; −0.034]	0.319^***^^b^	[0.262; 0.376]
Girls	−0.074^**^	[−0.123; −0.025]	−0.067^**^	[−0.115; −0.020]	−0.071^**^	[−0.124; −0.018]
Turkish	−0.038	[−0.085; 0.009]	−0.035	[−0.085; 0.016]	0.034	[−0.016; 0.085]
**Explained variances (*****R**^2^***)**						
National self-identification	0.088					
Perceived national fit (separated)	0.253					
Perceived national fit (integrated)	0.156					
Perceived national fit (assimilated)	0.110					

*Standardized regression coefficients (β) with 95% confidence intervals (CI) are reported. Identical superscripts indicate effects that did not significantly differ*.

*** *p < 0.001*,

** *p < 0.01*,

* *p < 0.05*,

∧ *p = 0.292*.

As shown also in Figure 1, we, moreover, consistently found that minority adolescents' perceived acceptance of the imagined culturally different peers positively related to the extent to which they perceived these peers to fit with the national identity. These findings confirm Hypothesis 2, namely that minority adolescents perceive better national fit of culturally different peers, when they perceive these peers to be socially accepted by other nationals. Specifically, we found that minority youth considered the separated, integrated or assimilated peers in the vignettes to fit with the national identity better, when they perceived most Belgian nationals to accept the separated, integrated or assimilated peer more, correspondingly. However, the perceived national fit of the integrated and the assimilated peer were equally contingent on perceptions of acceptance [Wald χ^2^_(1)_ = 0.043, *p* = 0.835], while the perceived national fit of the separated peer depended more strongly on perceived acceptance than the perceived national fit of the integrated or assimilated peer [Wald χ^2^_(1)_ = 5.796, *p* = 0.016]. These findings suggest that especially the perceived national fit of those who are most culturally different from the majority group depends on the perceived acceptance by other nationals.

### Contextualizing perceived fit and acceptance

**Figure 2:**
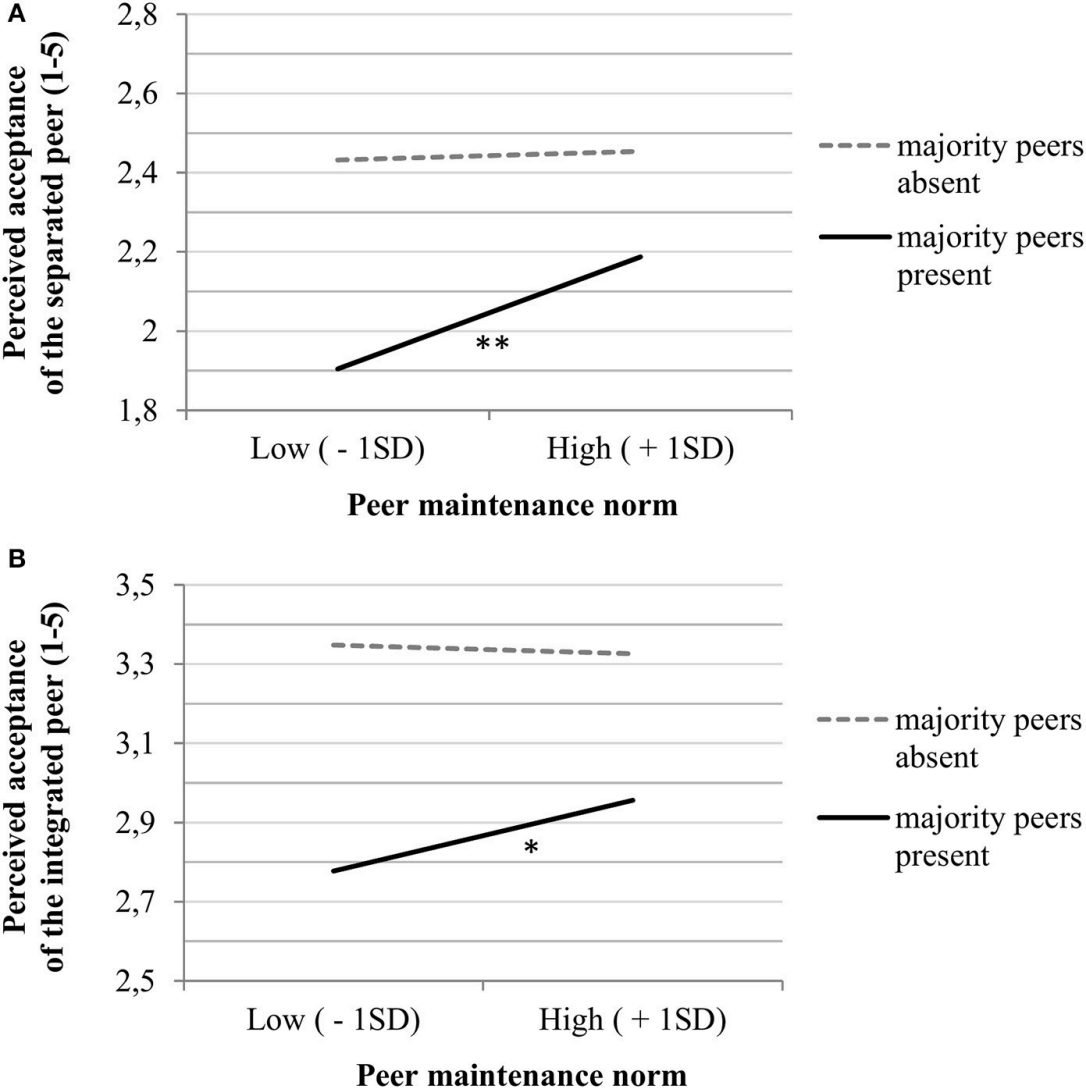
**(A)** Perceived acceptance of the (imagined) *separated* peer as a function of peer maintenance norm (hign vs. low) and the (lack of) majority peer presence in class ^**^p < 0.01. **(B)** Perceived acceptance of the (imagined) *intergrated* peer as a function of peer maintenance norm (hign vs. low) and the (lack of) majority peer presence in class ^*^p < 0.05.

The third and final research aim concerns the affordances of perceived national fit and acceptance by the actual day-to-day peer context in culturally diverse classrooms. To this end, we added the maintenance norm of the actual peers in class as a predictor of our measures of perceived acceptance, and ran a multi-level multi-group path model that distinguishes between minority participants who were in classrooms with a majority peer presence and those who were not. Here we focus specifically on the link between the actual peer norm of heritage culture maintenance and the perceived acceptance of the imagined culturally different peers (full model results available upon request). Regardless of a (lack of) majority peer presence, actual peer views on heritage culture maintenance were unrelated to minority adolescents' perceived acceptance of the imagined assimilated peer (no Figure shown). However, peer norms did matter for the extent to which Moroccan or Turkish minority youth perceived other Belgian nationals to accept the imagined more culturally different (separated or integrated) peers, but only among those in classrooms with a majority peer presence. As shown in Figures 2, a more positive actual peer norm of heritage culture maintenance in classrooms with a majority peer presence was positively associated with the perceived acceptance of the separated peer (β = 0.116, *p* = 0.002, 95% CI [0.043; 0.189]) and the integrated peer (β = 0.082, *p* = 0.010, 95% CI [0.019; 0.144]), respectively. No comparable significant associations were found for those in classrooms without majority peers. Although the average level of perceived acceptance of the separated and the integrated peers in the vignettes were somewhat higher for those in all-minority classrooms than those in classrooms with a majority peer presence, mean differences in perceived acceptance were not significant [separated: Wald χ^2^_(1)_ = 2.110, *p* = 0.146; integrated: Wald χ^2^_(1)_ = 2.080, *p* = 0.149]. To sum up, these results provide partial support for Hypothesis 3, namely that acculturation norms in diverse classrooms, in particular whether (actual) peers value the maintenance of a distinct heritage culture, inform the perceived acceptance of (imagined) peers who are culturally different from the majority. Since Hypothesis 3 did not hold for those in all-minority classrooms, our findings also show the role of majority peer presence for the actual view of peers to inform minority adolescents' perceived acceptance of culturally different peers.

### Indirect paths

Although no mediation hypotheses were formulated, we did test for indirect paths (using “model indirect” in Mplus 7). In our first model (see Figure 1 with indirect paths indicated in bold), we found significant indirect effects of the perceived acceptance of (imagined) culturally different peers on national self-identification via perceived national fit: The Moroccan or Turkish minority youth who perceived other Belgian nationals to be more accepting of the separated or integrated peer, also correspondingly perceived these peers as fitting the national identity better and these fit perceptions in turn positively predicted their national self-identification [separated: β_indirect_ = 0.035, *p* = 0.022, 95% CI [0.005; 0.066] and integrated: β_indirect_ = 0.025, *p* = 0.027, 95% CI [0.003; 0.048], respectively]. Moreover, in the subsequent model, we found that the peer norm of heritage culture maintenance in classrooms with a majority peer presence indirectly increased minority adolescents' perceived national fit of the imagined more culturally different (separated or integrated) peers by strengthening their perception that other Belgian nationals are accepting of them [separated: β_indirect_ = 0.054, *p* = 0.002, 95% CI [0.019; 0.088] and integrated: β_indirect_ = 0.030, *p* = 0.017, 95% CI [0.006; 0.055], respectively]. Taken together, these indirect effects provide first tentative empirical evidence for a context-dependent cognitive pathway that allows minority youth to perceive national fit and acceptance in the presence of cultural difference from the majority, and hence to belong as fellow nationals.

### Control variables

With regard to our control variables, we found that national self-identification was higher among girls than boys, and higher among Turkish than Moroccan minority youth. However, girls were less likely than boys to perceive the (imagined) culturally different peers to fit with the national identity. No difference in fit perceptions was found between Moroccan and Turkish minority youth. Finally, controlling for participants' own acculturation attitudes also made the assimilation-identification link visible in our models: Minority adolescents' national self-identification was positively related to their positive attitude toward majority culture adoption, and negatively related to their positive attitude toward heritage culture maintenance.

### Robustness checks

We carried out additional robustness checks. First, because the vignettes (see Table 1) specifically describe Turkish Muslim minority youth, we re-ran the analyses controlling for participants' *attitude toward Turks* (using the well-known “feeling thermometer” as a global measure of in-group or out-group feelings^6^). While positive attitudes toward Turks negatively predicted national self-identification, the associations of self-identification with perceived fit and acceptance were fully replicated (see Table S1).^7^

Second, for the same reason, we re-ran the analysis splitting the sample into Moroccan and Turkish subgroups (comparing baseline, fully constrained and partially constrained multi-group models). The comparative model fit in Table S2 confirms that the associations of interest were exactly replicated across the subgroups, except for one differential association [Satorra Bentler ΔChi^2^(5) = 6.367, *p* = 0.272]: Perceived acceptance was more strongly related to perceived national fit for the separated peer in the Moroccan than in the Turkish subgroup [Wald χ^2^_(1)_ = 6.473, *p* = 0.011]. Since the direction and the significance level (at *p* < 0.001) of this association did not differ across subgroups, however, we conclude that our findings are robust.

Moreover, because 17% of the minority youth in our sample did not self-identify as Muslim, we re-ran our analyses controlling for participants' *attitudes toward Muslims* (also measured with the well-known “feeling thermometer”). While a positive attitude toward Muslims negatively predicted national self-identification and the perceived fit of the integrated and separated peers in the vignettes, the associations of national self-identification with perceived fit and acceptance were again fully replicated (model results available upon request)^8^.

Together, these additional robustness checks replicate our findings regardless of ethnic or religious attitudes or differences. Therefore, and to ensure an optimal distribution of individual participants over contextual units (classroom) in our multi-level design, Moroccan and Turkish ethnic origin youth—regardless of their religious attitudes or differences—were pooled in the main analyses.

## Discussion

Much previous research has shown that minorities in Europe generally report lower national belonging than majorities (e.g., Fleischmann and Phalet, 2018). Not feeling fully “at home” in the country they live and often were born in negatively affects in particular the well-being and success of minority youth (Walton and Cohen, 2011). Against the backdrop of prevailing assimilationist views, the present study examined minority perceptions of the national fit and acceptance of acculturating peers who differ culturally from the majority (aim 1). Next, we examined the consequences of perceived national fit and acceptance for minority adolescents' own national belonging (aim 2); and, finally, we asked when perceived national fit and acceptance are afforded by the peer context in culturally diverse classrooms (aim 3). Focusing on Moroccan and Turkish minority youth in Belgium, we add to the research literature on the national identification of immigrant minorities by articulating a minority perspective on the intersubjective cultural contents of the national identity.

The first aim of this study was to establish minority perceptions of national fit and acceptance of culturally different peers. Our results clearly show that the Moroccan and Turkish minority youth were aware that national identity in Belgium is predominantly defined in majority cultural terms. As expected, minority participants perceived separated or integrated peers in the vignettes, who are more culturally different from the majority, to fit the national identity less well than the assimilated peer, who is least culturally different from the majority. Similarly, they also perceived other nationals to be least accepting of the separated peer and most accepting of the assimilated peer in the vignettes.

Our second research aim was to test the consequences of perceived fit and acceptance for minority adolescents' own national belonging. In line with our reasoning, we found that minority youth who perceived culturally different peers to fit the national identity better, self-identified more strongly as Belgian nationals. This finding suggests that national identity definitions that include those who are culturally different from the majority group facilitate minorities' national belonging. Specifically, we argued that perceptions of national fit in the presence of cultural difference from the majority enable minorities to mentally link their self-relevant heritage culture and the national group—analogous to the process of “self-anchoring” (Van Veelen et al., 2016) – and thus can strengthen their national belonging. This positive link between fit perceptions and own national belonging was independent of minority adolescents' own acculturation attitudes. We controlled for minority participants' attitude toward majority culture adoption and heritage culture maintenance, respectively, to simultaneously account for the well-documented assimilation-identification pathway via “self-stereotyping.”

Our study results, however, also reflect that minority youth as individuals can more readily define national identity to include those who are culturally different from the majority, when more inclusive understandings of national fit are socially recognized by other nationals (Verkuyten, 2005). Accordingly, Moroccan and Turkish minority adolescents' perceptions of national fit in our study depended on the extent to which they perceived other Belgian nationals to be accepting of culturally different peers. Especially the perceived national fit of the (imagined) most culturally different peer was contingent on perceived social acceptance. Moreover, the perceived acceptance of culturally different peers was indirectly related to minority adolescents' own national belonging, through strengthening their perception of the national fit of culturally different peers. These associations suggest that minority perceptions of national fit are closely entwined with how they perceive other nationals to accept peers who are culturally different from them.

The third and final research aim concerns the affordance of perceived national fit and acceptance by the peer context in culturally diverse classrooms. Here, two study findings stand out. First, we found that a majority peer presence in class was a necessary condition for peer norms to inform minority perceptions of how accepting other nationals are of culturally different peers. All-minority peer contexts are disconnected from the national majority group who powerfully define the national identity in the wider society; and, our results suggest that minority peers cannot define the national identity in the absence of social verification by majority peers. As minority youth in Belgium (as in other European countries) increasingly find themselves in classrooms with few or no majority peers (Baysu and de Valk, 2012; Celeste et al., 2016), future research should explore how minority youth in segregated classrooms form their perceptions of the fit and acceptance of culturally different peers.

Second and as expected, only in classrooms with a majority peer presence did minority adolescents perceive other Belgian nationals to be more accepting of the (imagined) culturally different peers when their actual peers valued minorities maintaining their distinct heritage culture. Moreover, they also perceived culturally different peers to fit better with the national identity than in classes where heritage culture maintenance was less valued. Thus, our study results imply that schools and teachers should promote inclusive acculturation norms among students in mixed classrooms to facilitate the national belonging of minority adolescents.

### Strengths, limitations and future directions

Social psychology research to date has paid little empirical attention to minority perspectives on the cultural contents of national identities in European migration contexts. Most social identity research on minority national identification has focused on the strength and structure of multiple national, ethnic and/or religious identities rather than on the meaningful contents of the national identity (cf. Verkuyten and Martinovic, 2012 for a review). Our theoretical interest was in how the perceived national fit and acceptance of (imagined) culturally different peers relate to minority adolescents' own national belonging. The present study adds to a rich research literature on the acculturation and identification of minority youth by focusing on the perceived intersubjective cultural *contents* of national identity as—related to, yet—distinct from the *strength* of national self-identification. We were specifically interested in whether perceived national identity contents refer exclusively to the majority culture, or whether they leave some room for contributions from minorities' distinct heritage cultures. Future studies may extend our research by focusing on specific cultural or other contents of the national identity in relation to minorities' self-identification as nationals.

Furthermore, a distinctive empirical strength of our study design is its strong external validity. We used a large-scale survey with stratified random samples of participants covering a wide range of real-life settings in a multi-level design. Due to its sampling design, our study includes minority adolescents in schools with very different levels of ethnic segregation: from all-majority to all-minority classrooms, thus ensuring optimal external validity, i.e., the generalizability of our findings to the wider population of minority youth across a wide range of real-life contexts. In today's societies and schools in Europe, settings with very few or no majority youth are increasingly common (Baysu and de Valk, 2012), yet little is known about the acculturation and identification of minority youth in these contexts. The design of our study enabled us to empirically test the contextual affordances of perceived national fit and acceptance among minority youth in classrooms with and without a majority peer presence (cf. supra). In addition, because we had contextual classroom measures complementing individual-level responses, we were able to assess the role of peer acculturation norms and majority peer presence in the actual peer context independently from minority students' own acculturation attitudes and perceptions.

Another strong point of our study is that vignettes were used to measure minority perceptions of the national fit of their peers in a subtle and unobtrusive way. We focused on the perceived national fit of imagined acculturating peers rather than on minority adolescents' own national fit to avoid self-representation biases; our vignettes allowed us to implicitly tap into shared understandings of the cultural contents of the national identity (Chiu et al., 2010) as distinct from minority adolescents' own perceived fit which may be motivated by their own belonging.

Because our vignettes were embedded in a large-scale survey of minority adolescents, there is, however, a trade-off in our study design between (strong) external and (limited) internal validity. Future research should follow up on this limitation by randomizing the order of the vignettes and by counterbalancing the names and gender of imagined peers with the cultural contents of the vignettes in more rigorous lab experiments. Moreover, our vignettes can be fine-tuned further by future lab experiments varying specific cultural features while controlling all other features.

Another limitation of this study is that we relied on cross-sectional data, and therefore cannot provide evidence for causality. Specifically, we analyzed perceived national fit of (imagined) culturally different peers as a predictor of minority adolescents' national self-identification, but there may also be reverse or reciprocal influences. Minorities might also project their own views onto others (Thijs and Verkuyten, 2016), so that they, for instance, perceive more acceptance of culturally different peers when they themselves view these peers to fit the national identity better. In support of our theoretical reasoning, however, our external contextual measure of peer acculturation norms in mixed (but not in all-minority) classrooms indirectly predicted minorities' perceptions of national fit. While the cross-sectional nature of our measures does not allow us to empirically decide causal direction, our findings suggest that minority youth in mixed classrooms where heritage culture maintenance is valued may infer that most nationals will accept minorities who maintain their heritage culture. As a consequence, they will sooner perceive peers who are culturally different from the majority as real nationals. Nevertheless, future research should extend the current analyses longitudinally as a more rigorous test of the proposed alternate pathway to national belonging from the contextual affordances of minority perceptions of national fit and acceptance.

Moreover, we acknowledge that our data allowed for only single-item measures. The construct validity of our measures is, however, supported by our tested models; associations, also those with control variables, are meaningful and in line with our theoretical expectations. Moreover, our measure of national self-identification has been used successfully in previous studies (e.g., Leszczensky et al., 2016; Fleischmann and Phalet, 2018), and research has shown that single-item measures can assess social identification adequately (Postmes et al., 2013). Still, it would be good for future research to use composite measures to replicate our findings.

Furthermore, our minority participants represent most devalued and culturally distant Muslim minorities in European societies, who are commonly perceived to misfit with the national identity and who report relatively low levels of national belonging across European countries (Fleischmann and Phalet, 2018). Hence, future studies should replicate the role of perceived national fit and acceptance for the national belonging of less stigmatized or more culturally similar minority groups, or in more welcoming and less assimilationist societies than the Belgian context.

Finally, our study shows that there is significant variation in minority adolescents' perceptions of the national fit and acceptance of peers who are culturally different from the majority. Specifically, our study findings suggest that minority youth in mixed peer contexts which value their distinct heritage cultures can define the national identity to include minority cultural references as well. We consider this is a key finding of the present study. Most research assumes that minorities adopt a prevailing majority definition of national identity, thus bypassing the essentially contested nature of national identity contents—even within the majority group as evident from research on citizenship representations (Reijerse et al., 2013). Our preliminary findings call for more systematic research on the meaningful contents of the national identity from the perspective of cultural minorities, and how these meanings are afforded by, for instance, local peer contexts.

## Conclusions

To conclude, the present study reveals an alternate cognitive pathway for minority youth to achieve national belonging, *when* they perceive culturally different peers as fitting the national identity and being accepted by other nationals. When Moroccan and Turkish minority adolescents in Belgium perceived culturally different peers to fit the national identity better, they also self-identified more strongly as Belgian nationals. At the same time, we show that minority adolescents' perceptions of national fit and acceptance depend on the peer context in culturally diverse classrooms. In the presence of majority peers, peer norms valuing heritage culture maintenance can facilitate the national belonging of minority youth through opening up the national identity to cultural difference from the majority. Future research should shed light on what informs minority adolescents' perceptions of national fit and acceptance in the segregated classrooms where majority peers are absent.

## Ethics statement

This study is part of a multi-study research program titeld TRACES (TRjectories of Acculturation and Contact in Ethnically diverse Social networks), which has been approved by Social and Societal Ethics Committee, University of Leuven, Belgium. Written informed consent was obtained from the parents/legal guardians of all participants.

## Author contributions

NG developed the study concept. KP provided the data which NG analyzed and interpreted with input from the other authors. NG and KP drafted several versions of the paper and FF added her critical comments and suggestions. All authors approved the final version of the manuscript.

### Conflict of interest statement

The authors declare that the research was conducted in the absence of any commercial or financial relationships that could be construed as a potential conflict of interest.

## Footnotes

^1^For a discussion of the various benefits (and/or costs) of integration, other than the promotion of personal self-esteem, please see the review paper by Nguyen and Benet-Martínez (2013).

^2^1st generation = born in Morocco or Turkey, 2nd generation = at least one parent born in Morocco or Turkey, 3rd generation = no parent, but at least two grandparents born in Morocco or Turkey.

^3^Similar age and gender distributions were given across the two sub-groups: (1) Moroccan (*N* = 834), *M*_age_ = 15.04 (*SD* = 1.18), 47% female; (2) Turkish (*N* = 655), *M*_age_ = 15.04 (*SD* = 1.18), 47% female.

^4^More specifically, response categories for our measure of national self-identification were (1) “I do not feel Belgian,” (2) “not strongly,” (3) “not so strongly,” (4) “strongly,” and (5) “very strongly.”

^5^Associations across vignettes were, for instance, those of the perceived acceptance of the (imagined) separated peer with the perceived fit of the (imagined) integrated and assimilated peers, respectively.

^6^Specifically, we asked “Please rate how you feel about Turks in Belgium on a scale that runs from 0 to 100. The higher the number, the more positive you feel. The lower the number, the more negative you feel toward this group”. Participants responded on an 11-point scale with 10° increments in-between (0 = very negative, 100 = very positive feelings toward Turks).

^7^We also re-ran our analysis controlling for *religious salience* (measured as the subjective importance of religion) as yet another individual-level covariate. While religious salience negatively predicted national self-identification, the associations of nationals self-identification with perceived fit and acceptance were fully replicated (model results available upon request).

^8^In addition, we ran our analysis with and *without* including the subgroup of Moroccan or Turkish minorities who did not self-identify as Muslim (17%). The results of our main analysis were fully replicated even when excluding those who did not self-describe themselves as Muslims (model results available upon request).
