# Supplementary material for: Contingent National Belonging: The Perceived Fit and Acceptance of Culturally Different Peers Predicts Minority Adolescents' Own Belonging
Source: Front Psychol. 2018 Oct 29;9:1975. doi: 10.3389/fpsyg.2018.01975 (PMC6215841; doi:10.3389/fpsyg.2018.01975)
Supplement: Supplementary file 1 [file Table_1.pdf]

Table S1. Robustness Check 1: Including *own attitude towards Turks* as an additional control in the multi-level path model with national self-identification as the final dependent variable and while controlling for students nested in school classes

**Effects on national self-identification**	β	CI				
Perceived national fit (separated)	0.072 * [a]	[0.008; 0.136]				
Perceived national fit (integrated)	0.072 * [a]	[0.011; 0.133]				
Perceived national fit (assimilated)	- 0.029 ^	[- 0.087; 0.029]				
Own attitude towards maintenance	- 0.085 **	[- 0.144; - 0.027]				
Own attitude towards adoption	0.222 ***	[0.163; 0.282]				
Own attitude towards Turks	- 0.072 *	[- 0.142; - 0.002]				
Girls	0.107 ***	[0.049; 0.165]				
Turkish	0.120 ***	[0.055; 0.186]				
**Effects on perceived national fit**	**separated**		**integrated**		**assimilated**	
	β	CI	β	CI	β	CI
Perceived acceptance (separated)	0.471 ***	[0.420; 0.521]	0.074 *	[0.012; 0.136]	- 0.056	[- 0.116; 0.004]
Perceived acceptance (integrated)	0.025	[- 0.034; 0.084]	0.360*** [b]	[0.297; 0.424]	- 0.009	[- 0.072; 0.054]
Perceived acceptance (assimilated)	- 0.125 ***	[- 0.179; - 0.071]	- 0.085 **	[- 0.140; - 0.031]	0.318 *** [b]	[0.261; 0.375]
Own attitude towards Turks	- 0.031	[- 0.100; 0.038]	- 0.026	[- 0.097; 0.045]	0.003	[- 0.070; 0.076]
Girls	- 0.073 **	[- 0.122; - 0.023]	- 0.066 **	[- 0.114; - 0.018]	- 0.071 **	[- 0.124; - 0.018]
Turkish	- 0.027	[- 0.079; 0.025]	- 0.026	[- 0.080; 0.029]	0.033	[- 0.023; 0.089]
**Explained Variances (R^2)**						
National self-identification	0.098					
Perceived national fit (separated)	0.253					

Perceived national fit (integrated)	0.156
Perceived national fit (assimilated)	0.109

Notes. Standardized regression coefficients (β) with 95% confidence intervals (CI) are reported. Identical superscripts indicate effects that did not significantly differ. *** $p < 0.001$, ** $p < 0.01$, * $p < 0.05$, ^ $p = 0.329$
