# Supplementary material for: Contingent National Belonging: The Perceived Fit and Acceptance of Culturally Different Peers Predicts Minority Adolescents' Own Belonging
Source: Front Psychol. 2018 Oct 29;9:1975. doi: 10.3389/fpsyg.2018.01975 (PMC6215841; doi:10.3389/fpsyg.2018.01975)
Supplement: Supplementary file 2 [file Table_2.pdf]

Table S2. Robustness Check 2 – Replication across Moroccan and Turkish origin subgroups: Comparative model fit of unconstrained, fully and partially constrained multi-level multi-group path models

	Number of free parameters	Loglikelihood	SCF	SB $\Delta Chi^2(df)$
*Un*constrained model (baseline)	101	- 25135.568	1.2696	
Fully constrained model (model 1) (imposing equality constraints on all 6 theoretical associations across groups)	95	- 25144.180	1.2730	14.167(6), p = 0.028
Partially constrained model (model 2) (freely estimating 1 differential association across groups)	96	- 25139.341	1.2740	6.367(5), p = 0.272

Notes. Model 1 imposes equality constraints on the associations: (1) of perceived national fit in separated, integrated and assimilated vignettes with national self-identification, and (2) of perceived acceptance with perceived national fit in the same vignettes. Model 2 freely estimates only the association of perceived acceptance with fit for the separated vignette, which was stronger for the Moroccan than for the Turkish group (Wald $\chi^2(1) = 6.473$, p = 0.011). SCF = Scaling Correction Factor. SB = Santorra Bentler.
